# Is discard better than return gastric residual aspirates: a systematic review and meta-analysis

**DOI:** 10.1186/s12876-019-1028-7

**Published:** 2019-06-28

**Authors:** Zunjia Wen, Ailing Xie, Mingqi Peng, Lanzheng Bian, Li Wei, Mei Li

**Affiliations:** Children’s Hospital of Nanjing Medical University, No.72 Guangzhou road, Gulou district, Nanjing, Jiangsu province China

**Keywords:** Gastric residual volume, Enteral nutrition, Nursing care, Management, Review, Meta-analysis

## Abstract

**Background:**

The assessment of residual gastric volume is common practice in critical care units. However, the effects and safety of discarding or returning gastric aspirates remain uncertain. Therefore, we aimed to evaluate the role of discarding or returning gastric aspirates on the gastric residual volumes in critically ill patients.

**Methods:**

A comprehensive, systematic meta-analysis of randomized controlled trials (RCTs) on the efficacy and safety of discarding or returning gastric aspirates in critical ill patients was performed. Studies were identified by searching Pubmed and other databases (from inception to 31 Sept 2018). Summary odd ratios (ORs) or mean differences (MDs) with 95% confidence intervals were calculated using fixed- or random-effects model for outcome assessment.

Results: Four RCTs, with a total number of 314 adult patients, were included in the analysis. No significant differences were found in the 48th hour residual volume (MD = 8.89, 95% CI: 11.97 to 29.74), the average potassium level (MD = 0.00, 95% CI: − 0.16 to 0.16), the episodes of gastric emptying delay (OR = 0.98, 95% CI: 0.35 to 2.80), the incidence of aspiration pneumonia (OR = 0.93, 95% CI: 0.14 to 6.17), the episodes of nausea or vomiting (OR = 0.53, 95% CI: 0.07 to 4.13) and diarrhea (OR = 0.99, 95% CI: 0.58 to 1.70).

**Conclusions:**

No evidence confirms that returning residual gastric aspirates provides more benefits than discarding them without increasing potential complications. Rigorously designed, multi-center, large-sample randomized controlled trials must be further conducted to validate the role of discarding or returning residual gastric aspirates.

## Background

Nutritional support is an essential part of patient management in intensive care unit (ICU) [1]. In general, a common practice of health care providers is to insert a nasogastric or orogastric tube to provide nutrition support, and nutrition delivery by the gastrointestinal tract is more economic and physiological than the parenteral route; enteral nutrition (EN) helps maintain the structure and function of intestinal mucosa, reduce the risk of infection, and avoid the potential adverse effects of parenteral nutrition (PN) [2–4]. Critically ill ICU patients with feeding tube are at a high risk for many complications, such as gastric retention, pulmonary aspiration, and feeding intolerance, considering their impaired consciousness level, unstable physiological status, and intervene of mechanical ventilation [5, 6]. Therefore, scholars must explore methods for maximizing the benefits and reducing the complications of feeding tube for nutrition support to improved outcomes of critical ill patients.

Monitoring gastric residual volume (GRV) is typically conducted to observe signs of feeding intolerance. Previous studies [7–9] have investigated the use of GRV to observe gastric tolerance during EN, the results remain inconsistent, and many clinicians [10, 11] speculate that monitoring GRV is unnecessary. Moreover, the ideal cutoff for intervention and frequency of monitoring GRV has not been established yet [12]. Further investigations must be conducted to guide the clinical practice. To date, several studies have focused on management of gastric aspirates; their results on whether to return or discard gastric aspirates remain controversial. Despite a certain number of randomized controlled trials (RCTs) on management of gastric aspirates, the variability of intervention time, sample size, and outcomes remains large. In this regard, critical review of related studies and synthesize of data of RCTs are necessary to provide evidence for management of gastric aspirates and insights into future direction on this issue. This systematic review and meta-analysis aims to evaluate whether discarding or returning gastric aspirates can improve the outcomes of ICU patients with feeding tube.

## Methods

We conducted this systematic review in compliance with the recommendations from Cochrane Collaboration and reported results in accordance with preferred reporting items for systematic reviews and meta-analyses (PRISMA) [13, 14].

### Data sources and search strategies

Two authors independently searched the databases for appropriate studies. Related articles were identified and selected by searching in Pubmed, EMBASE, Web of Science, Science Direct, Cochrane Central Register of Controlled Trials, China National Knowledge Infrastructure, Wanfang Database, and the Chinese Biomedical Literature Database (from inception to 31 August 2018) with the following search terms: (nasogastric tube OR gastric tube OR gastric feeding OR enteral nutrition OR EN) AND (gastric residual volume OR GRV OR gastric residual aspirate OR aspirate) AND (discard OR return or management). Searching was conducted through strategies used in Pubmed and the instructions and rules of each database. The reference lists of the retrieved studies and previous reviews and meta-analyses were manually search for potential RCTs. No language limits were set for the identification of related publications.

### Study selection

Studies were selected on the basis of the first screening of the identified titles or abstracts and the second check-up of full-text articles. Studies were considered eligible if the following criteria were met: the study design was RCT; the study subjects included critical ill adult patients with feeding tube; the intervention was to return or discard gastric aspirates with orogastric tube; we made no restriction on the timing of starting and ending of the intervention; and related outcomes, such as GRV, gastric emptying delay, aspiration pneumonia, and feeding intolerance, were reported and data could be retrieved. Case reports, series, qualitative studies, and review articles were excluded. All studies were screened independently by the two authors (Z W and A X) according to the selection criteria. The studies were selected when the two reviewers consented. Any disagreement on the studies for inclusion was settled with resort to a third reviewer (M L).

### Data extraction

A standardized data collection form was designed to extract important information. Any discrepancies in the extraction process were resolved by consents. We also attempted to contact authors to obtain original data or missing details. Two reviewers (Z W and A X) independently extracted the following information: first author, year of publication, study location, target population, details of discarding and returning residual gastric aspirates, main outcomes, and conclusions.

The following outcomes were extracted: the primary outcome was the gastric residual volume: the volume of gastric residuals at different time-point after the first return or discard of the gastric residual. The second outcomes included the blood test of electrolyte level, and related complications including the number of patients with episodes of gastric emptying delay, aspiration pneumonia, vomiting, diarrhea.

### Quality assessment

Two reviewers independently used Cochrane Collaboration’s risk of bias tool to evaluate the methodological quality and risk of bias of the included RCTs. This tool includes seven specific domains: sequence generation, allocation concealment, blinding of participants and personnel, blinding of outcome assessment, incomplete outcome data, selective outcome reporting, and other issues. Each domain is classified as low risk of bias, high risk of bias, or unclear risk of bias according to the judgment criteria. Furthermore, we assessed the quality of evidence using the GRADE criteria [15]. Any disagreements in the quality assessment were resolved by discussion and consents.

### Data synthesis and analysis

All of the extracted data were processed and analyzed with Review Manager version 5.3 [16]. Data were extracted and double-checked by two reviewers independently. Data syntheses and interpretations were performed by two authors (Z W and M P) to ensure the accuracy of the results. Binary outcomes were presented as Mantel–Haenszel-style odds ratios (ORs) with 95% confidence intervals (CIs). Continuous outcomes were reported as mean differences (MDs). A fixed-effect model was adopted in case of homogeneity (*P*-value of χ^2^ test > 0.10 and *I*^2^ < 50%). A random-effect model was used in case of significant heterogeneity (P-value of χ^2^ test > 0.10 and *I*^2^ ≥ 50%). We planned to do subgroup analysis if there are evident heterogeneity, and the source of heterogeneity was analyzed according to patients’ age, the threshold of discarding or returning gastric residuals among trials. Publication bias was evaluated with funnel plots, and asymmetry was assessed by conducting Egger regression test. Differences at *P* < 0.1 were considered statistically significant.

## Results

**Fig. 1:**
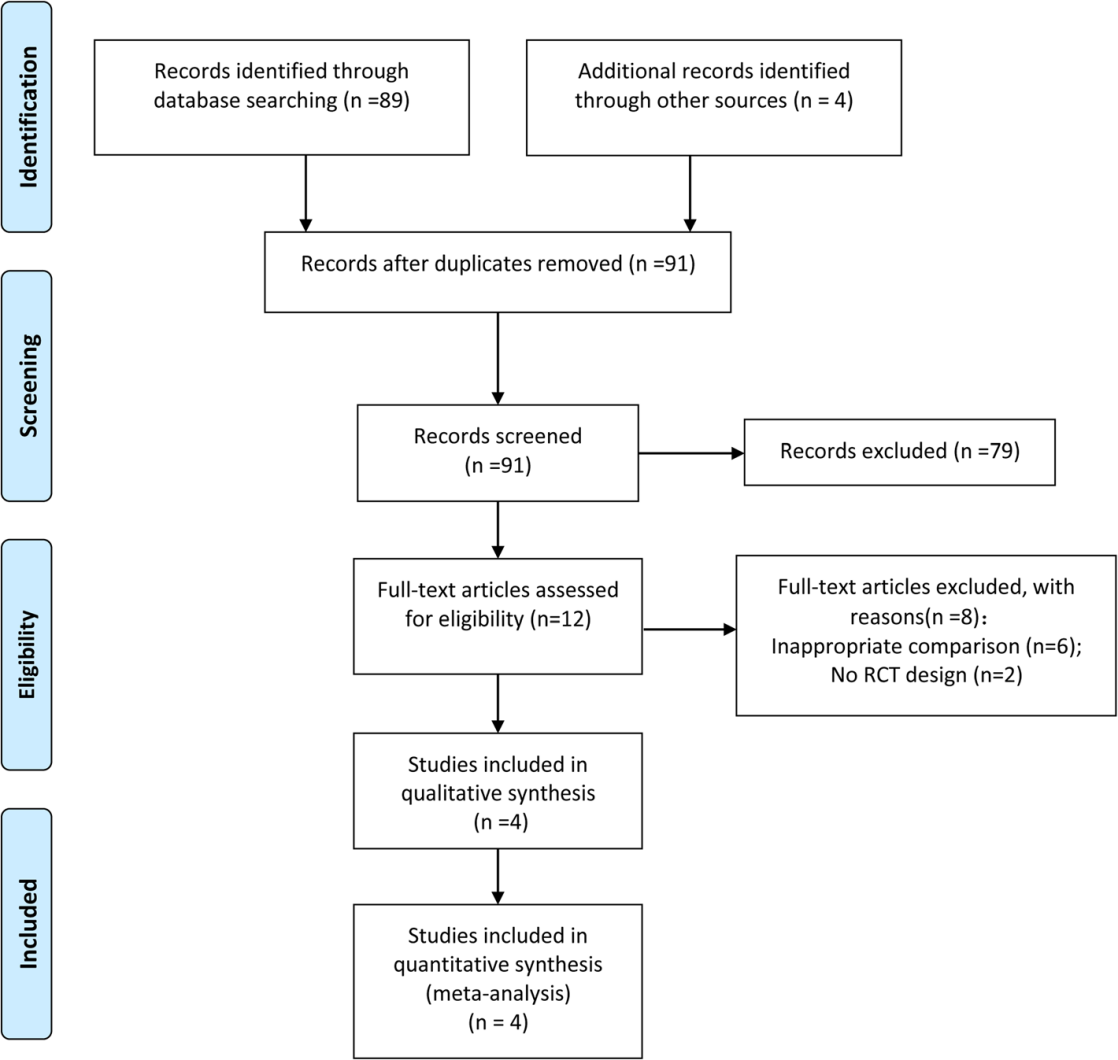
Flow chart of study selection

Figure 1 indicates the PRISMA flow chart of study selection. Briefly, 91 relevant publications were identified through the initial comprehensive search. After screening the titles and abstracts of the citations, we included 12 potentially related studies for another round of full-text review. These studies were further screened and subjected to quality appraisal. We then included four RCTs [17–20] for final analysis.

### Study characteristics

Table 1 shows the basic characteristics of the four included RCTs, which involved 314 adult patients, comprising 156 patients who underwent discard intervention and 158 patients who underwent return intervention. All of the four RCTs were conducted in different countries, namely, Egypt [19], the USA [18], Spain [17], and China [20]. The follow-up period varied from 2 days [17, 18] to 7 days [19]. Two RCTs [17, 19] returned gastric aspirates up to 250 mL, one RCT [20] returned gastric aspirates up to 150 mL, and one RCT [18] returned all gastric aspirates. Three RCTs [17–19] reported the number of patients with episodes of gastric emptying delay in discard and return groups, and the delayed gastric emptying is reckoned as there are more GRV than the nominated GRV in those studies.

**Table 1:** The characteristics of included studies

Study (author year)	Country	Sample (discard /return)	Population	Intervention		Outcomes	Conclusions
				Discard group	Return group		
Behairy 2014 [19]	Egypt	44 (20/24)	Adult patients connected with EN within first 24 h and for 7 consecutive days	Discarded all gastric aspirate before feeding,	Returned the gastric aspirate up to 250 ml	The GRV, gastric emptying delay, the aspiration pneumonia, feeding intolerance (vomiting & diarrhea), the electrolytes & glucose level, comfort outcomes (vital signs and oxygen saturation) on 1st and 7th day.	It is recommended to return gastric aspirate up to 250 ml to the patients.
Booker 2000 [18]	USA	18 (10/8)	Critically ill adult ICUs patients with expected EN > 48 h.	Discarded all the residual volumes before feeding	Had all the residuals returned through the feeding tube.	Weight changes; serum level of electrolytes; complications such as diarrhea, nausea, vomiting et al.	It’s tempting to encourage nurses to discard the residual volumes.
Juvé-Udina 2009 [17]	Spain	122 (61/61)	Critically ill ICU adult patient with estimated length of stay > 48 h	Any aspirate was discarded.	Returned the gastric aspirate up to 250 ml	Nasal gastric tube obstructive complication episodes; pulmonary aspiration episodes; intolerance episodes (nausea, vomiting, diarrhoea and abdominal distension); enteral feeding delays; hyperkalaemia, hyperglycaemia episodes; discomfort episodes	Re-introduce gastric content aspirated to improve GRV management is favored
Wang 2017 [20]	China	130 (65/65)	Surgical ICU adult patients with total or part EN	All the aspirates were discarded.	Returned the gastric aspirate up to 150 ml	The incidence of gastric emptying delay; the serum level of blood sugar, potassium, blood sodium; related complications (the incidence of gastric retention, tube blockage, diarrhea and aspiration)	Re-transfusion of gastric retention fluid is recommened.

### Risk of bias assessment

**Fig. 2:**
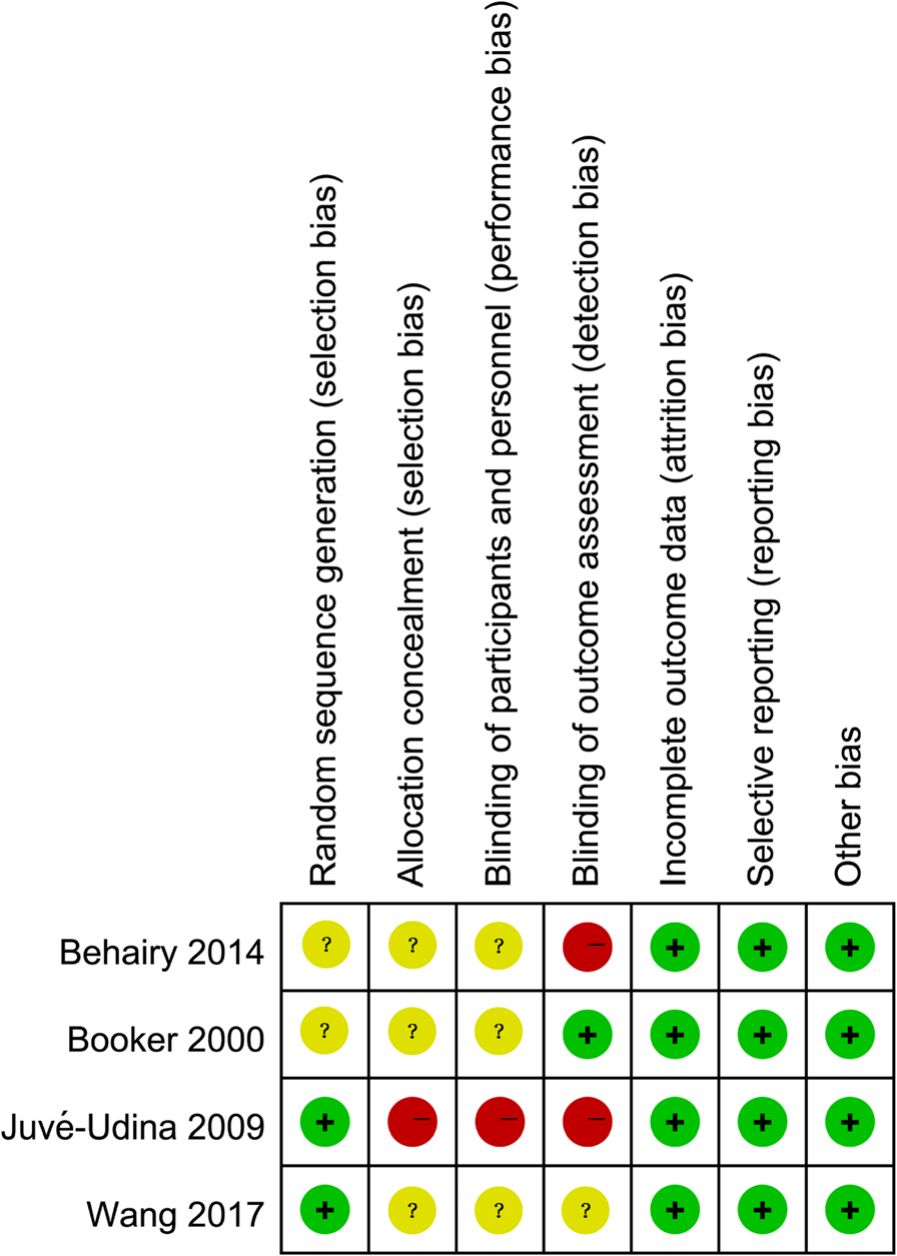
Risk of bias graph

**Fig. 3:**
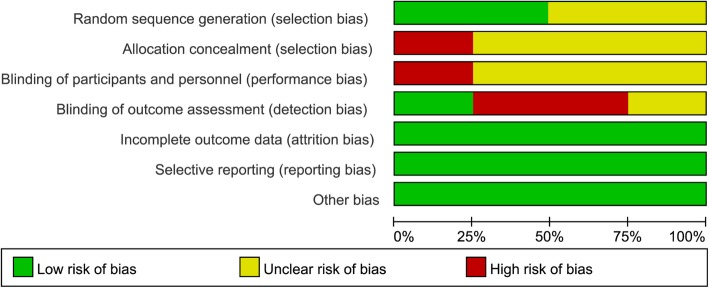
Risk of bias summary

Figures 2 and 3 represent the risk of bias of included in the four RCTs. All of the RCTs mentioned the randomization, yet the method of randomization of two RCTs [18, 19] remained unclear, and none reported allocation blinding. Significant exposure of allocation was observed in one RCT [17], whereas the allocation concealment of resting three RCTs [18–20] remained unclear. Given the nature of interventions, blinding participants and personnel is difficult; therefore, no study reported the blinding design on participants and personnel. Only one RCT [18] was rated with low risk of bias considering their blinding design on the assessment process. No other significant biases were found among the included RCTs.

### Outcome analysis

#### Forty-eight hour residual volume

**Fig. 4:**
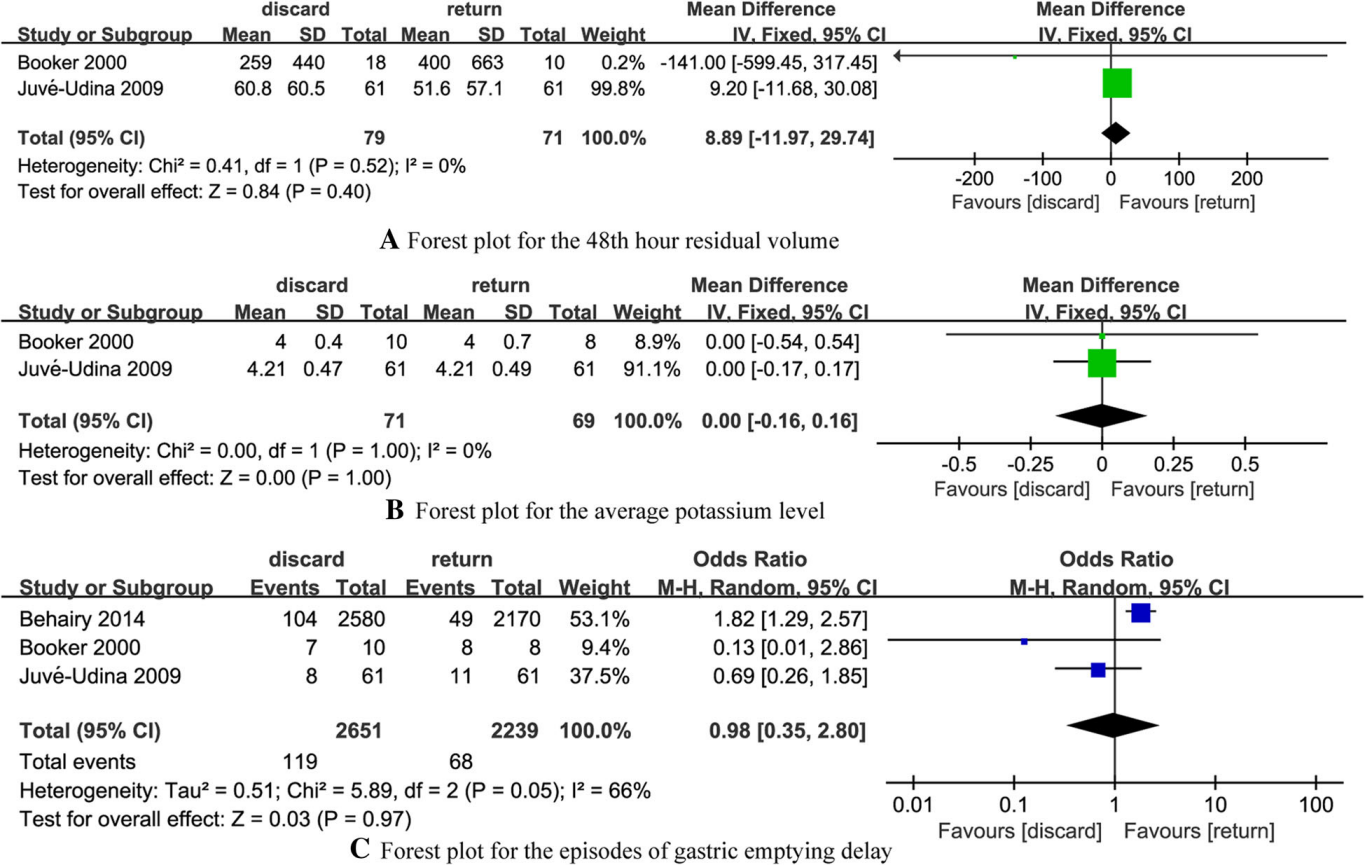
Forest plot for different outcomes. **a** Forest plot for the 48th hour residual volume. **b** Forest plot for the average potassium level. **c** Forest plot for the episodes of gastric emptying delay

Two RCTs [17, 18] reported the 48th hour residual volume in discard and return groups. The summary MD on the 48th hour residual volume was 8.89 (95% CI: − 11.97 to 29.74), and no evident heterogeneity was found (*P* = 0.52, *I*^2^ = 0%) (Fig. 4a).

#### Average potassium level

Two RCTs [17, 18] reported the average potassium level in discard and return groups. The summary MD on the average potassium level was 0.00 (95% CI: − 0.16 to 0.16), and no evident heterogeneity was found (*P* = 1.00, *I*^2^ = 0%) (Fig. 4b).

#### Episodes of gastric emptying delay

Three RCTs [17–19] reported the episodes of gastric emptying delay in discard and return groups. The summary OR on the episodes of gastric emptying delay was 0.98 (95% CI: 0.35 to 2.80), and heterogeneity was evident (*P* = 0.05, *I*^2^ = 66%) (Fig. 4c).

#### Incidence of aspiration pneumonia

**Fig. 5:**
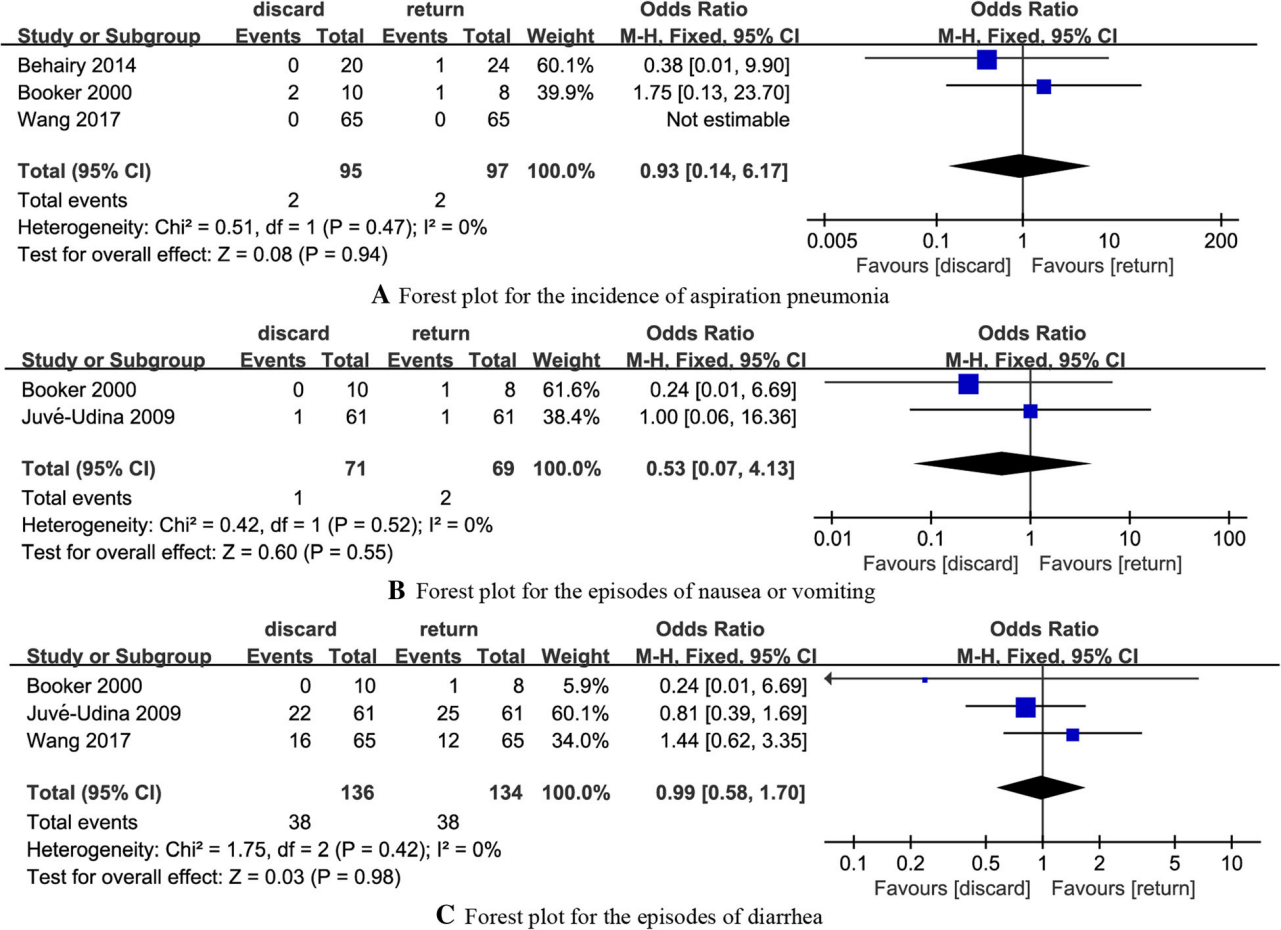
Forest plot for different outcomes. **a** Forest plot for the incidence of aspiration pneumonia. **b** Forest plot for the episodes of nausea or vomiting. **c** Forest plot for the episodes of diarrhea

Three RCTs [18–20] reported the incidence of aspiration pneumonia in discard and return groups. The summary OR on the incidence of aspiration pneumonia was 0.93 (95% CI: 0.14 to 6.17), and no evident heterogeneity was found (*P* = 0.47, *I*^2^ = 0%) (Fig. 5a).

#### Episodes of nausea or vomiting

Two RCTs [17, 18] reported the episodes of nausea or vomiting in discard and return groups. The summary OR on the episodes of nausea or vomiting was 0.53 (95% CI: 0.07 to 4.13), and no evident heterogeneity was found (*P* = 0.52, *I*^2^ = 0%) (Fig. 5b).

#### Episodes of diarrhea

Three RCTs [17, 18, 20] reported the episodes of diarrhea in discard and return groups. The summary OR on the episodes of diarrhea was 0.99 (95% CI: 0.58 to 1.70), and no evident heterogeneity was found (*P* = 0.42, *I*^2^ = 0%) (Fig. 5c).

### Subgroup and sensitivity analyses

No subgroup analyses were performed in our study because of the small heterogeneity and limited number of the included RCTs. We attempted to evaluate publication bias by using a funnel plot. However, publication bias was not determined because of the limited number of the included RCTs (less than 10).

Sensitivity analyses are used to investigate the influence of one study on the overall risk estimate by removing one study in each turn for every included results. The results suggested that the overall risk estimates were not substantially changed by any single study.

## Discussion

To return or discard gastric residual volume is an important question that warrants discrete verification. Gastric residues may increase the risk of tube blockage and infection, whereas discarding gastric residues may increase the risk of fluid and electrolyte imbalance in patients [21, 22]. The three included RCTs [17, 19, 20] indicated that the return of gastric residues provided more benefits on the balance of fluid and electrolyte but did not increase the gastric intolerance; these studies supported the return of gastric residues to improve the outcomes of critically ill adult patients. However, our synthesized results indicate no significant differences in the 48th hour residual volume, the average potassium level, the episodes of gastric emptying delay, the incidence of aspiration pneumonia, and the episodes of vomiting and diarrhea for adult patients. Even when including a larger sample size, we still did not detect differences in the benefits of discarding and returning residual gastric aspirates, and the quality of evidence was generally moderate (Table 2). A previous systematic review and meta-analysis [23] in Chinese patients included two RCTs [17, 18]; consistent with our present finding, this work did not provide evidence that returning gastric residual aspirates can effectively improve the management of gastric retention without increasing potential complications. Therefore, more rigorous, multi-center, large-sample RCTs are needed to validate the effects of discarding or returning residual gastric aspirates and provide solid evidence and guidance to clinical practice.

**Table 2:** The summary of synthesized findings

Outcomes	Number of included studies	Number of participants ontributing data to this outcome	Summary MD/OR	95% CI	Heterogeneity (I^2^)	Quality of evidence (GRADE)
Forty-eight hour residual volume	2	150	8.89	−11.97 to 29.74	0%	⊕ ⊕ ⊕ O Moderate
Average potassium level	2	140	0.00	−0.16 to 0.16	0%	⊕ ⊕ ⊕ O Moderate
Episodes of gastric emptying delay	3	4890	0.98	0.35 to 2.80	66%	⊕ ⊕ ⊕ O Moderate
Incidence of aspiration pneumonia	3	192	0.93	0.14 to 6.17	0%	⊕ ⊕ ⊕ O Moderate
Episodes of nausea or vomiting	2	140	0.53	0.07 to 4.13	0%	⊕ ⊕ O O Low
Episodes of diarrhea	3	170	0.99	0.58 to 1.70	0%	⊕ ⊕ ⊕ O Moderate

At present, the amount of gastric residues to be taken as gastric retention has not been clearly defined yet. Based on literature review, most studies [9] regarded gastric retention as 150–400 mL, but the values ​​vary greatly. A computer simulation of nutrient solution infusion and gastric emptying was performed using a combination of nine gastric emptying speeds and six infusion rates; the results showed that for healthy people, if intestinal feeding is performed at 0–125 mL per hour, then the gastric residue will be rapidly stabilized at 225–900 mL at the beginning of enteral feeding for 3 to 13 h [24]. That is, different critically ill patients have different levels of gut dysfunction dependent on their pathology, shock states or therapies applied that reduce/impair gut motility, spanning from 225 mL to 900 mL. Therefore, the numerical range of gastric retention in different populations must be determined. Previous studies [10, 25] reported that for children, if the gastric residual amount exceeds 5 mL/kg, then gastric emptying delay and gastric retention occur. However, one previous study [26] has found that the bedside EN intolerance assessments, particularly GRV, do not predict delayed GE or rate of EN advancement. One of our included studies [19] has divided gastric emptying delay into three levels based on an amount of more than 150 mL: 151–250 mL as light delay, 251–350 mL as moderate delay, and over 350 mL as severe delay; however, the return of residual gastric aspirates was not evaluated based on the severity of gastric emptying delay. The return of residual gastric aspirates should be based on the discrete consideration of gastric retention degree [27]. With greater gastric residual volume, the return of gastric residual aspirate is suspected to cause higher risks in gastric retention. Two of the included RCTs [17, 19] returned gastric aspirates up to 250 mL, one RCT [20] returned gastric aspirates up to 150 mL, and one RCT [18] returned all gastric aspirates. Future studies should address the advantages and disadvantages of GRV monitoring to potentially identify the cutoffs of GRV in different populations and provide recommendations on return of residual gastric aspirates.

The potential advantages and disadvantages of discarding and returning residual gastric aspirates should be considered. Discarding aspirates may reduce the handling of feed delivery systems for medical staff and the risk of potential contamination, but it exposes staff to splash injury [27]. However, whether discarding residual gastric aspirates reduces the level of gastric retention remains unclear. In the present meta-analysis, the result of the 48th hour residual volume indicate no difference in discarding and returning aspirates; the result is similar to that of the 7th day residual volume [19]. Discarding residual gastric aspirates may result in insufficient nutritional supplement for the patient and higher risk of fluid and electrolyte disturbance [28]. Meanwhile, returning residual gastric aspirates can improve the management of nutrition delivery and balance of fluid and electrolyte; however, this process is prone to higher risk of tube blockage and contamination [9, 29]. It’s well-known that discarding the residual gastric aspirates can increase the risk of reducing energy intake, however, the very abnornal looking aspirates such as bloody, fecal or very bilious aspirates are virtually always discarded since it’s a sign of gastric bleeding or intolerance [30]. The present study did not found any difference between the return and discard intervention in terms of the average potassium level, the episodes of gastric emptying delay, and related adverse complications. The discard or return of residual gastric aspirates should be highly individualized and appropriately used after careful assessment of the potential benefits and risks of such therapy, considering specific short- and long-term goals.

This meta-analysis has several limitations that should be considered. First, although this study did not found effect and safety differences between discard and return intervention, the rather small sample size of the pooled studies may be insufficient to detect significant difference. Given the limited number and outcome differences, subgroup analyses and funnel plot evaluation were not conducted. Second, some of the included RCTs recruited patients who received EN and PN. PN may result in impaired bowel function, and its inclusion may confuse the results. Further evidence should be obtained using a rigorous standardized nutrition regimen to investigate the effects and safety of discarding or returning aspirates in patients with EN or PN. Third, the randomization procedures are well explained in all of the included RCTs. However, the process of allocation, blindness, and assessment is unclear, although it is essential to the adequacy and reliability of the results, we have attempted to contact the original authors to get the related information on their own studies, yet no reply has been received. Future studies with rigorous design and large sample sizes are warranted to identify the role of discarding and returning residual gastric aspirates.

## Conclusions

In conclusion, this meta-analysis failed to determine effect and safety differences between discarding or returning residual gastric aspirates. The optimal strategy for management of residual gastric aspirates in critically ill patients has yet to be determined. To date, management of GRV and residual gastric aspirates varies greatly. Limited lines of evidence are available to guide the practice, especially in the population of children. Further studies on this area must be conducted to evaluate the role of discarding or returning residual gastric aspirates.

## Data Availability

All data generated or analyzed during this study are included in this published article.

## Abbreviations

CIs: Confidence intervals
EN: Enteral nutrition
GRV: Gastric residual volume
ICU: Intensive care unit
MDs: Mean differences
ORs: Odd ratios
PRISMA: Preferred reporting items for systematic reviews and meta-analyses
RCTs: Randomized controlled trials

## References

[1] Zhang H, Wang Y, Jiang ZM, Kondrup J, Fang H, Andrews M, Nolan MT, Mu SY, Zhang J, Yu K (2017). Impact of nutrition support on clinical outcome and cost-effectiveness analysis in patients at nutritional risk: a prospective cohort study with propensity score matching. Nutrition.

[2] Parent BA, Seaton M, Djukovic D, Gu H, Wheelock B, Navarro SL, Raftery D, O'Keefe GE (2017). Parenteral and enteral nutrition in surgical critical care: plasma metabolomics demonstrates divergent effects on nitrogen, fatty-acid, ribonucleotide, and oxidative metabolism. J Trauma Acute Care Surg.

[3] Senussi NH (2017). Enteral nutrition in the treatment of Crohn’s disease: overlooked and underutilized. Am J Gastroenterol.

[4] Lewis SR, Schofield-Robinson OJ, Alderson P, Smith AF (2018). Enteral versus parenteral nutrition and enteral versus a combination of enteral and parenteral nutrition for adults in the intensive care unit. Cochrane Database Syst Rev.

[5] McClave SA, DeMeo MT, DeLegge MH, DiSario JA, Heyland DK, Maloney JP, Metheny NA, Moore FA, Scolapio JS, Spain DA (2002). North American summit on aspiration in the critically ill patient: consensus statement. JPEN J Parenter Enteral Nutr.

[6] Zhu Y, Yin H, Zhang R, Ye X, Wei J (2018). Gastric versus postpyloric enteral nutrition in elderly patients (age >/= 75 years) on mechanical ventilation: a single-center randomized trial. Crit Care.

[7] Kuppinger DD, Rittler P, Hartl WH, Ruttinger D (2013). Use of gastric residual volume to guide enteral nutrition in critically ill patients: a brief systematic review of clinical studies. Nutrition.

[8] Hsu CW, Sun SF, Lee DL, Lin SL, Wong KF, Huang HH, Li HJ (2011). Impact of disease severity on gastric residual volume in critical patients. World J Gastroenterol.

[9] Montejo JC, Minambres E, Bordeje L, Mesejo A, Acosta J, Heras A, Ferre M, Fernandez-Ortega F, Vaquerizo CI, Manzanedo R (2010). Gastric residual volume during enteral nutrition in ICU patients: the REGANE study. Intensive Care Med.

[10] Tume LN, Bickerdike A, Latten L, Davies S, Lefevre MH, Nicolas GW, Valla FV (2017). Routine gastric residual volume measurement and energy target achievement in the PICU: a comparison study. Eur J Pediatr.

[11] Ozen N, Tosun N, Yamanel L, Altintas ND, Kilciler G, Ozen V (2016). Evaluation of the effect on patient parameters of not monitoring gastric residual volume in intensive care patients on a mechanical ventilator receiving enteral feeding: a randomized clinical trial. J Crit Care.

[12] Elke G, Felbinger TW, Heyland DK (2015). Gastric residual volume in critically ill patients: a dead marker or still alive?. Nutr Clin Pract.

[13] Knobloch K, Yoon U, Vogt PM (2011). Preferred reporting items for systematic reviews and meta-analyses (PRISMA) statement and publication bias. J Craniomaxillofac Surg.

[14] Moher D, Liberati A, Tetzlaff J, Altman DG, Group P (2009). Preferred reporting items for systematic reviews and meta-analyses: the PRISMA statement. J Clin Epidemiol.

[15] Brożek J. L., Akl E. A., Alonso-Coello P., Lang D., Jaeschke R., Williams J. W., Phillips B., Lelgemann M., Lethaby A., Bousquet J., Guyatt G. H., Schünemann H. J. (2009). Grading quality of evidence and strength of recommendations in clinical practice guidelines. Allergy.

[16] Wen Z, Shen M, Wu C, Ding J, Mei B. Chewing gum for intestinal function recovery after caesarean section: a systematic review and meta-analysis. BMC pregnancy and childbirth. 2017;17(1):105. 10.1186/s12884-017-1286-8 PMC5394625 28415967

[17] Juve-Udina ME, Valls-Miro C, Carreno-Granero A, Martinez-Estalella G, Monterde-Prat D, Domingo-Felici CM, Llusa-Finestres J, Asensio-Malo G (2009). To return or to discard? Randomised trial on gastric residual volume management. Intensive Crit Care Nurs.

[18] Booker KJ, Niedringhaus L, Eden B, Arnold JS (2000). Comparison of 2 methods of managing gastric residual volumes from feeding tubes. Am J Crit Care.

[19] Behairy AS, Elsedawy ED (2014). Effect of returning versus discarding gastric aspirate on the occurrence of gastric complications and comfort outcomes on enteral feeding patients. J Nat Sci Res.

[20] Wang L, Chen J, Zou M (2017). Influence of infusion of gastric fluid retention on gastric remnant and its compl ications in critical ICU patients. Chin Nurs Res.

[21] Ahmad S, Le V, Kaitha S, Morton J, Ali T (2012). Nasogastric tube feedings and gastric residual volume: a regional survey. South Med J.

[22] Tume LN, Latten L, Kenworthy L (2017). Paediatric intensive care nurses’ decision-making around gastric residual volume measurement. Nurs Crit Care.

[23] Wang L, Cheng Y, Zou M, Hu Y (2014). Systematic review on the reinfusion of gastric retention fluid for nasal feeding patients. Chin Nurs Res.

[24] Lin HC, Van Citters GW (1997). Stopping enteral feeding for arbitrary gastric residual volume may not be physiologically sound: results of a computer simulation model. JPEN J Parenter Enteral Nutr.

[25] Horn D, Chaboyer W, Schluter PJ (2004). Gastric residual volumes in critically ill paediatric patients: a comparison of feeding regimens. Aust Crit Care.

[26] Martinez EE, Pereira LM, Gura K, Stenquist N, Ariagno K, Nurko S, Mehta NM (2017). Gastric emptying in critically ill children. JPEN J Parenter Enteral Nutr.

[27] Williams TA, Leslie GD (2010). Should gastric aspirate be discarded or retained when gastric residual volume is removed from gastric tubes?. Aust Crit Care.

[28] Williams TA, Leslie G, Mills L, Leen T, Davies H, Hendron D, Dobb GJ (2014). Frequency of aspirating gastric tubes for patients receiving enteral nutrition in the ICU: a randomized controlled trial. JPEN J Parenter Enteral Nutr.

[29] Williams TA, Leslie GD (2004). A review of the nursing care of enteral feeding tubes in critically ill adults: part I. Intensive Crit Care Nurs.

[30] Poveda VB, Castilho A, Nogueira LS, Ferretti-Rebustini REL, Silva R (2018). Assessing gastric residual volume: a description of nurses’ clinical practice. Rev Esc Enferm USP.

